# Dataset on the change of postharvest quality of *Physalis peruviana* L. as an effect of ethylene inhibitor

**DOI:** 10.1016/j.dib.2019.103849

**Published:** 2019-03-20

**Authors:** Syariful Mubarok, Salma Dahlania, Nursuhud Suwali

**Affiliations:** Department of Agronomy, Faculty of Agriculture, Universitas Padjadjaran, Bandung, 45363 Indonesia

## Abstract

Ethylene will be a major problem in postharvest quality of fresh fruit such as *Physalis peruviana* L. that belongs to an important medicinal plant. This data article describes the alteration of fruit quality of *P. peruviana* L. during 21 days of postharvest storage as an effect of ethylene inhibitor, 1-Methylcyclopropene (1-MCP), at different concentration and duration of application. Nutritional parameters and fruit shelf life from 1-MCP treated fruit with three level concentrations (0.5, 1.0, and 2.0 μL L^−1^) and three level of durations application (6, 12 and 24 hours) were analyzed.

**Table:** Specifications table

Subject area	*Agriculture*
More specific subject area	*Postharvest, Horticulture*
Type of data	*Table, figure*
How data was acquired	*Ultrasonic Hardness Tester, SevenCompact* pH*/Ion S220, Refractometer PAL-J*
Data format	*Analyzed*
Experimental factors	*Yellowish green berry in color with greenish to pale yellow calyx were harvested to be treated with different 1-MCP treatment. This experiment consisted of nine treatments: (combination from three of 1-MCP concentration: 0.5, 1.0 and 2.*0 μL L ^*−1*^*and three of 1-MCP duration exposure: 6, 12 and 24 hours) and control repeated third times.*
Experimental features	*Determination of fruit shelf life, and other fruit quality i.e. fruit firmness, TA, TSS and p*H
Data source location	*Sumedang, Indonesia.*
Data accessibility	*The data are available with this article and accessible to the public.*
Related research article	*Olivares-Tenorio* et al. *[1]**and Balaguera-López* et al. *[2]*

**Table:** 

**Value of the data** • The data indicate the 1-MCP at the appropriate concentration and duration have a beneficial effect in improving postharvest life of P. peruviana L. • Data could be used by the researchers to make more understanding the potential used of 1-MCP for improving postharvest life of P. peruviana L. • Data could be used as an initial or basic data for further experiment in P. peruviana L.

## 1 Data

Data were represented the postharvest fruit quality of *P. peruviana* L. as an effect of various 1-MCP concentrations and exposure durations. Several parameter related to the postharvest quality, namely fruit shelf life, total soluble solid (TSS), fruit firmness, fruit pH and titratable acidity (TA) were analyzed. Table 1 presented data on fruit shelf life as the effect of concentration and duration of 1-MCP. Table 2 presented data on fruit firmness. Table 3 presented data on TSS. Table 4 presented data on fruit pH. Table 5 presented data on fruit TA.

**Table 1:** Fruit shelf life of *P. peruviana* L. at different concentration and duration of exposure of 1-MCP.

Treatments	Fruit Shelf Life (Days)
Control	17.0^a^
0.5 μL L^−1^, 6 h	21.3^c^
0.5 μL L^−1^, 12 h	21.7^c^
0.5 μL L^−1^, 24 h	20.7^bc^
1.0 μL L^−1^,6 h	21.7^c^
1.0 μL L^−1^, 12 h	21.0^bc^
1.0 μL L^−1^, 24 h	20.0^bc^
2.0 μL L^−1^, 6 h	20.0^bc^
2.0 μL L^−1^, 12 h	20.3^b^
2.0 μL L^−1^, 24 h	20.7^b^

Note: No significant differences are detected in mean value followed by same alphabetic annotation according to Duncan's Multiple Range Test (DMRT) at p < 0.05.

**Table 2:** Fruit firmness of *P. peruviana* L. at different concentration and duration of exposure of 1-MCP during 21 days of postharvest storage.

Treatments	Fruit Firmness (kgf)			
	0 DAS	7 DAS	14 DAS	21 DAS
Control	4.33^a^	3.52^a^	3.10^a^	2.73^a^
0.5 μL L ^−1^, 6 h	4.39^a^	4.20^d^	3.44^bc^	3.22^cd^
0.5 μL L ^−1^, 12 h	4.69^a^	3.75^ab^	3.43^bc^	3.33^d^
0.5 μL L ^−1^, 24 h	4.36^a^	4.17^cd^	3.29^ab^	3.06^bc^
1.0 μL L ^−1^,6 h	4.26^a^	3.74^ab^	3.22^ab^	3.18^cd^
1.0 μL L ^−1^, 12 h	5.04^a^	3.88^bc^	3.65^c^	3.02^abc^
1.0 μL L ^−1^, 24 h	4.82^a^	3.68^ab^	3.22^ab^	2.84^ab^
2.0 μL L ^−1^, 6 h	4.57^a^	3.64^ab^	3.30^ab^	2.87^ab^
2.0 μL L ^−1^, 12 h	4.85^a^	3.78^ab^	3.33^ab^	2.98^abc^
2.0 μL L ^−1^, 24 h	4.96^a^	3.74^ab^	3.33^ab^	3.08^bc^

Note: No significant differences at the same storage time are detected in mean value followed by same alphabetic annotation according to Duncan's Multiple Range Test (DMRT) at p < 0.05. DAS = days after storage.

**Table 3:** TSS of *P. peruviana* L. at different concentration and duration of exposure of 1-MCP during 21 days of postharvest storage.

Treatments	TSS (^o^Brix)			
	0 DAS	7 DAS	14 DAS	21 DAS
Control	14.10^a^	15.07^cd^	15.60^d^	14.93^b^
0.5 μL L ^−1^, 6 h	14.20^a^	14.60^a^	15.13^a^	15.50^de^
0.5 μL L ^−1^, 12 h	14.00^a^	14.70^ab^	15.17^a^	15.50^de^
0.5 μL L ^−1^, 24 h	13.60^a^	14.87^bc^	15.23^a^	14.77^b^
1.0 μL L ^−1^,6 h	12.60^a^	15.00^cd^	15.33^abc^	15.27^cd^
1.0 μL L ^−1^, 12 h	14.20^a^	15.07^cd^	15.30^abc^	15.17^c^
1.0 μL L ^−1^, 24 h	14.10^a^	15.03^cd^	15.27^ab^	14.53^a^
2.0 μL L ^−1^, 6 h	14.10^a^	15.30^d^	15.47^bcd^	14.90^b^
2.0 μL L ^−1^, 12 h	14.20^a^	15.23^d^	15.47^bcd^	15.43^de^
2.0 μL L ^−1^, 24 h	14.00^a^	15.00^cd^	15.50^cd^	15.53^e^

Note: No significant differences at the same storage time are detected in mean value followed by same alphabetic annotation according to Duncan's Multiple Range Test (DMRT) at p < 0.05.. DAS = days after storage.

**Table 4:** Fruit pH of *P. peruviana* L. at different concentration and duration of exposure of 1-MCP during 21 days of postharvest storage.

Treatments	Fruit pH			
	0 DAS	7 DAS	14 DAS	21 DAS
Control	5.1^a^	5.17^a^	5.30^a^	5.67^a^
0.5 μL L ^−1^, 6 h	5.1^a^	5.13^a^	5.17^a^	5.47^a^
0.5 μL L ^−1^, 12 h	5.1^a^	5.10^a^	5.13^a^	5.37^a^
0.5 μL L ^−1^, 24 h	5.0^a^	5.03^a^	5.20^a^	5.57^a^
1.0 μL L ^−1^,6 h	5.2^a^	5.27^a^	5.30^a^	5.40^a^
1.0 μL L ^−1^, 12 h	5.2^a^	5.20^a^	5.30^a^	5.60^a^
1.0 μL L ^−1^, 24 h	5.1^a^	5.13^a^	5.23^a^	5.57^a^
2.0 μL L ^−1^, 6 h	5.2^a^	5.23^a^	5.27^a^	5.43^a^
2.0 μL L ^−1^, 12 h	5.2^a^	5.30^a^	5.37^a^	5.60^a^
2.0 μL L ^−1^, 24 h	5.1^a^	5.17^a^	5.27^a^	5.67^a^

Note: No significant differences at the same storage time are detected in mean value followed by same alphabetic annotation according to Duncan's Multiple Range Test (DMRT) at p < 0.05.. DAS = days after storage.

**Table 5:** Titratable acidity of *P. peruviana* L. at different concentration and duration of exposure of 1-MCP during 21 days of postharvest storage.

Treatments	Titratable Acidity (%)			
	0 DAS	7 DAS	14 DAS	21 DAS
Control	1.86^a^	1.74^a^	1.54^a^	1.19^bc^
0.5 μL L ^-1^, 6 h	1.86^a^	1.76^a^	1.57^a^	1.48^d^
0.5 μL L ^-1^, 12 h	2.20^a^	1.89^bc^	1.77^a^	1.48^d^
0.5 μL L ^-1^, 24 h	2.50^a^	1.91^cd^	1.61^a^	1.31^cd^
1.0 μL L ^-1^,6 h	2.49^a^	1.96^c^	1.68^a^	1.24^bc^
1.0 μL L ^-1^, 12 h	2.33^a^	1.96^cd^	1.62^a^	0.94^a^
1.0 μL L ^-1^, 24 h	1.81^a^	1.78^ab^	1.73^a^	1.31^cd^
2.0 μL L ^-1^, 6 h	2.15^a^	2.07^d^	1.68^a^	1.30^cd^
2.0 μL L ^-1^, 12 h	1.86^a^	1.76^a^	1.67^a^	1.07^ab^
2.0 μL L ^-1^, 24 h	1.81^a^	1.75^a^	1.72^a^	1.08^ab^

Note: No significant differences at the same storage time are detected in mean value followed by same alphabetic annotation according to Duncan's Multiple Range Test (DMRT) at p < 0.05.. DAS = days after storage.

## 2 Experimental design, materials, and methods

### 2.1 Fruit preparation

The sample of *P. peruviana* L. fruits were obtained and harvested from Waida Farm in Sumedang, West Java, Indonesia. Similar fruit maturation at mature green (MG) fruits were chosen to be harvested according Baumann and Meier [3] and Trinchero et al. [4] with the criteria of yellowish green berry in color with greenish to pale yellow calyx and harvested from the same plant. Harvested fruit was kept at ambiance temperature (23 ± 4 °C) and 80% of humidity for postharvest quality analysis. The experiment consisted of nine treatments: (combination from three of concentrations and three exposure duration application) and three control replicates.

### 2.2 Fruit shelf life

The shelf life is one the important fruit character for *P. peruviana* L. fruit. It was counted from the initial day of storage when the fruit was still yellowish green berry in color with greenish to pale yellow calyx, up until quality lost characteristics were detected such as yellowish orange in color and flesh softens [3].

### 2.3 Fruit firmness

Fruit firmness was assayed in accordance to Mubarok et al. [5]. Briefly, four fruits in each replication were penetrated on two opposite side of the equatorial axes of fruit using a hand penetrometer of Ultrasonic Hardness Tester (Nippon Optical Works, Tokyo, Japan). Fruit firmness was measured for 7 until 21 days after storage (DAS).

### 2.4 Total soluble solid (TSS)

Sugar content were estimated by the value of TSS. TSS was measured every 7 days until 21 DAS based on method described in Mubarok et al. [5]. Briefly, *P. peruviana* L. fruit were blended and centrifugated for 10 mins at 13,000 × *g*. Obtained supernatant were used to determine TSS by using PAL-J refractometer (Atago, Tokyo, Japan).

### 2.5 Fruit TA and pH

The TA and pH were measured every 7 days until 21 DAS according to methods described by Garner et al. [6] and Dalal et al. [7] with modification. pH meter (Mettler-Toledo AG, Schwerzen-bach, Switzerland) was used to determine pH value from fruit juice. For TA analysis, briefly, 10 g of fresh fruit were homogenized by 100 mL distilled water and centrifugated for 10 mins at 13,000 × *g*. The supernatant was titrated with NaOH 0.1 N until pH reached 8.1. TA was represented as percentage of citric acid and calculated with the following equations:

$$\%\;TA=\left(V_{\text{NaOH}}\;x\;N_{\text{NaOH}}\;x\;0.064x100\right)/V_{\text{sample}}$$
